# *In situ* Creation of the Natural Phenolic Aromas of Beer: A Pulsed Electric Field Applied to Wort-Enriched Flax Seeds

**DOI:** 10.3389/fbioe.2020.583617

**Published:** 2020-10-19

**Authors:** Evangelia A. Tsapou, George Ntourtoglou, Fotini Drosou, Panagiotis Tataridis, Thalia Dourtoglou, Stavros Lalas, Vassilis Dourtoglou

**Affiliations:** ^1^Department of Wine, Vine, and Beverage Sciences, School of Food Science, University of West Attica, Athens, Greece; ^2^Department of Food Science and Nutrition, University of Thessaly, Karditsa, Greece

**Keywords:** ferulic acid, flax seeds, pulsed electric field (PEF), 4-vinylguaiacol (4-VG), *Saccharomyces cerevisiae*, non-*Saccharomyces*, yeast strain

## Abstract

To fine tune the production of phenolic aromas in beer, a pulsed electric field (PEF) was applied to beer wort, which was enriched with flax seeds. The choice of flax seeds as a source of FA is based on its high content of ferulic precursors and their intrinsic nutritional value. PEF was applied to ground flax seeds, with and without beta glycosidase. Fermentation was carried out with *Saccharomyces* and non-*Saccharomyces* yeast strains. Moreover, 4-vinylguaiacol (4-VG), a flavor highly active derived from volatile phenol, was produced by decarboxylation of ferulic acid (FA), or its precursor and flavor-inactive (4-hydroxy-3-methoxycinnamic acid). All yeast strains could metabolize FA into 4-VG, using the pure compound in the synthetic medium or in flax seeds, with the best quantity produced by *Saccharomyces cerevisiae* as a precursor. The method yields 4-VG production efficiencies up to 120% (mgL^−1^). Experimental treatment conditions were conducted with E= 1 kV/cm, total time treatment 15 min (peak time t_i_ = 1 μs, pause time t_p_ = 1 ms, Total pulses 900^3^). Treatment efficacy is independent of the fermentation yeast.

## Introduction

Flavoring with natural compounds is well-known in the food and beverage industry, driven by consistent consumer demand for more “natural” foods. Industries can improve the techniques of extraction or isolation of aromatic compounds by frequently applying two or more technologies to isolate target molecules, thereby rendering the final aroma more attractive. However, the most difficult issue is to empower the typical aromatic character without changing the final product (from the commonly accepted taste and aroma). This is done by hydrolyzing enzymatically-bound aromatic compounds in polymeric precursors with glycolytic enzymes (i.e., in wine), by adding aromatic compound precursors or using physicochemical methods (e.g., distillation and enrichment of the beverage with the heads of distillates).

Beer as a complex beverage contains many flavor-active compounds, as brewers try to reach an appropriate balance of desired aromas and to avoid off-flavors. Phenolic compounds are always present in the final product; they can be extracted from grains and hops during the process of mashing or brewing. Some phenolic compounds have a small impact on beer, while others may cause some desirable or undesirable effects (Lentz, 2018).

Moreover, 4-vinylguaiacol (4-VG) is an important constituent in bier (Tressl et al., 1976), distilled spirits (whiskey, rum) (Lee et al., 2000), contributing to the overall sweet smoky background. Found in tobacco, roasted peanuts, traminer wines is considered to be an important aroma contributor. Conventional *Saccharomyces cerevisiae* brewing yeasts ferment beers, in which a clove-like aroma is desired (Vanbeneden and Gils, 2008; Goncalves et al., 2016). Despite being undesirable in bottom-fermented beers, 4-VG is a well-known, positive aroma compound found in top-fermented beers, including those brewed with unmalted wheat for Belgian white beers, as well as those brewed with malted wheat for German Weizen and Rauch beers. However, in most of the top-fermented blond and dark beers, this volatile compound's presence is essential for overall flavor perception (Goncalves et al., 2016). Hence, it is important for flavor perception in the best beers (Zhu and Cui, 2013; Mertens et al., 2017).

Ferulic acid (FA) is the precursor of 4-VG and is found in plant cell walls in its free form or is covalently bonded to the biopolymers (Johnson et al., 2000; Strandås et al., 2008; Tanruean and Rakariyatham, 2016).

During this work we attempt to reach phenolic flavor enhancement, avoiding off-flavors by a combination of two technologies applied to beer wort-enriched with flax seeds. The target compound was 4-VG. These applied technologies are summarized below:

- In addition to the wort, rich FA derivative precursors (contained in flax seeds) and hydrolysis of the covalently bonded precursor from natural polymers was handled by the application of β-glucosidase from almonds, with a wide substrate spectrum of C5 and C6 monosaccharides and oligosaccharides. The industrial extension would be by applying a β-glycosidase from a microbial source.
- Extraction of the precursor, assisted by PEF.

PEF is a relatively new technique and when applied for extraction, it disorganizes the plant or microbial cell by disrupting membranes and releasing cell metabolites from the inner to outer part of the cell (Yang et al., 2016). The induced electric fields destroy the membrane of microbial or plant cells, thus leading to complex phenomena from cell restructuration to cell death (Zeng et al., 2008; Delsart et al., 2012; Zhang et al., 2012; Bozinou et al., 2019). PEF has been applied to other crops of industrial interest, such as grapes, onions, potatoes, etc. It was mainly used as a non-thermal treatment of liquid foods, to inactivate microorganisms (Grahl and Markl, 1996; Alvarez et al., 2003). Other researchers introduced electric field treatment to accelerate the aging of young wine, due to extraction of flavor compounds from wood (Zeng et al., 2008; Drosou et al., 2017) or to improve the phenolic recovery, as extraction pre –treatment in beer (Martin-Garcia et al., 2020).

To increase the amount of FA in the final product, a combination of a natural precursor (raw material) (rich in FA, such as flax seed), and an enzymatic and electrotechnique were used before fermentation. To demonstrate the versatility of the technique, four strains of yeast were used: *S. cerevisiae* and three other non-*Saccharomyces*. The technique was applied in a synthetic medium or in beer wort.

## Materials and Methods

### Chemicals

Dichloromethane, chloroform, methanol, pentane (95%), diethyl ether (95%), and anhydrous sodium sulfate were purchased from Chem Lab (Athens, Greece). All reagents were of analytical quality: β-glycosidase and 3-octanol were used as an internal standard, while all chemicals used for medium A were purchased from Sigma Aldrich (St. Louis, MO, USA).

### Fermentation

- Yeast strains

Four yeasts strains (*S. cerevisiae* and three non-*Saccharomyces* yeast strains) were used in a concentration of 100 mgL^−1^ for each fermentation: *S. cerevisiae* US-05 (Fermentis), (SC) *Toluraspora delbrueckii* Prelude (Hansen), (Prelude) *Toluraspora delbrueckii* Biodiva 291 (Biodiva) (Lallemand), and *Metschnikowia pulcherrima* (Lallemand) (Mets).

- Batch of synthetic medium A supplied with pure FA

Synthetic medium A for main cultures, inoculated with 0.1 gL^−1^ lyophilized microorganisms, was prepared by dissolving KH_2_PO_4_ 1 gL^−1^; K_2_HPO_4_ 1 gL^−1^ (NH_4_)_2_SO_4_ 2 gL^−1^; MgSO_4_·7H_2_O 0.2 gL^−1^; ZnSO_4_·5H_2_O 0.2 gL^−1^; glucose 9 gL^−1^; maltose 86 gL^−1^; fructose 5 gL^−1^; (pH 5). Media were prepared in deionized water that day and autoclaved.

For media containing FA, pure FA is added to each sample at 12 mg for 150 mL (48 mgL^−1^). The volume of each fermentation was 150 mL for each yeast strain. Samples were kept at room temperature for 7 days to conduct yeast fermentation. All fermentations were performed in duplicate.

- Batch in Synthetic medium A supplied with flax seeds

Both varieties of flax seeds (gold and brown) were purchased from a local Greek market (Athens, Greece). Two fermentation bottles (150 mL) with cells were cultured batch-wise on synthetic medium A for each mentioned yeast strain. Both varieties of flax seeds (brown and gold) ground in a plate mill were added to the bottled after being crushed, for a quantity of 4 g per bottle. As such, 625 μL of a prepared solution of 4 gL^−1^ β-glucosidase from almonds (lyophilized powder, ≥ 2 units/mg solid) at pH 7.4 was added to each vial. This was left for 30 min for enzyme activation. As stated, all samples were incubated at 35°C for 7 days and conducted in duplicate.

- Flax seeds added to wort for beer production, plus PEF and Control fermentations, with and without β-glucosidase from almonds

Wort production:

➢ Mashing process: 1 kg of Pils malt (Macedonian Thrace Brewery S.A., Athens, Greece) ground in 1–1.2 mm and mixed with water at 55°C. The program was: 1°C min^−1^ up to 63°C to remain for 1 h. We find 1°C min^−1^ up to 72°C, remaining for approximately 15 min, and 1°C min^−1^ up to 78°C for 5 min. The process of brewing in a bag (BIAB) was used, and after the mash rest, the bag was attached to drain above the kettle and then squeezed.
➢ Sparging step: 1 L of water at 77–80°C was used for sparging during the mash rest, and again after squeezing the grain bag.
➢ Boiling process: The last bag was boiled for 90 min and the wort was used under sterilized conditions without adding hops. Wort was added in sterilized fermentable bottles (48 in total) at 60 mL, with 1.6 g of each variety of flax seed added to the bottles (16 with gold flax and 16 with brown flax).

PEF was applied in 16 substrates in duplicate (8 worts with gold flax and 8 worts with brown flax). The rest of the bottles (8 with gold flax and 8 with brown flax) were used as Control substrates without PEF treatment.

All substrates were inoculated with the same four yeast strains. At that point, 250 μL of the prepared solution of β-glucosidase was added to a bottle of each yeast and the other was used as the Control enzyme and for PEF fermentation. All samples were incubated at 35°C for 7 days.

### Pulsed Electric Field Procedure

The PEF equipment used was described previously by Ntourtoglou et al. (2020). The treatment chamber (TC) consisted of two rectangular flat stainless steel electrodes measuring 10 by 10 cm in size, separated by Teflon bars. The distance between the two electrodes was 1 cm. The electric field strength E was evaluated as E = U/d, where “U” is the applied voltage and “d” is the distance between two electrodes (d = 10 mm). In each case, treatment was calculated as: [***t*** **=** **(t**_**i**_ **+** **t**_**p**_**)** **×** **P**], t_i_ = peak time duration (μs), t_p_ = pause time (ms) and *P* = number of pulses. Experimental treatment conditions were conducted with E= 1 kV/cm, *t* = 15 min (t_i_ = 1μs, t_p_= 1 ms, 900^3^ pulses).

### Sample Preparation for GC-MS

In addition, 40 mL of each sample was mixed with organic solvents (mixture of 20 mL of pentane and 20 mL of diethyl ether) for 10 min at room temperature. The samples were centrifuged at 3,500 rpm for 10 min to separate the phases (Hermle Z200A, Milan, Italy). The supernatant was extracted a second time, using the same volume of solvent for 10 min. The organic layer was washed with distilled water in a separation funnel, while the organic phase was dried over anhydrous sodium sulfate and filtered. Samples were condensed in a flash evaporator and compressed with nitrogen until they were dry weight. Finally, 100 μL of dichloromethane was added to samples, from which 1.0 μL was used for GC–MS analysis. A concentration of 4-VG was calculated using a standard curve for Control and PEF fermentation, while the ones with synthetic medium 10 μL of 3-octanol (2,500 ppm) were added as an internal standard after being filtered.

### Gas Chromatography-Mass Spectrometry Analysis

Each sample was subjected to GC, coupled with MS analysis, using an Agilent 6890 series GC System (Wilmington, DE, USA), described by Ntourtoglou et al. (2020). All data were recorded with the Turbomass 5.0 ChemStation software (Agilent).

### Statistical Analysis

Statistical analysis for the standard deviation (SD) of the means and *t*-tests were carried out with Excel 2013 (Microsoft, Redmond, WA, USA).

## Results and Discussion

### Experiments in the Presence of Free FA or Flax Seeds: A Source of FA in Synthetic Medium A

As shown in Table 1, none of the four yeast strains was capable of producing 4-VG without the external addition of free FA or flax seeds to the synthetic medium A. When free FA was included in medium A, all strains of yeast demonstrated biotransformation of FA into 4-VG.

**Table 1:** 4VG formation by yeast strains cultivated in synthetic Medium A with and without the presence of FA, or the presence of flax seeds.

**4VG**	**Medium A without FA**		**Medium A** **+** **48 mg/L of FA**		**Medium A** **+** **26,7 g/L of flax seeds**	
	**Average**	**Efficacy [%]**	**Average**	**Efficacy [%]**	**Gold**	**Brown**
*S.C*.	n.d.^*^	n.d	31.81 ± 14.85	66.27	0.732	0.077
*Prelude*	n.d	n.d	11.38 ± 6.70	23.71	0.086	0.079
*Biodiva*	n.d	n.d	14.91 ± 4.83	31.06	0.083	0.000
*Mets*	n.d	n.d	22.04 ± 9.50	45.92	0.285	0.003

* *n.d, not detected*.

Three important conclusions are based on the findings:

1. *Saccharomyces* and non-*Saccharomyces* are strains with high exhibited FA decarboxylase activity. *S. cerevisiae* brewing yeasts contain active ferulate decarboxylase enzymes that can transform trans-FA into 4-VG. The enzyme responsible for the decarboxylation of FA is FDC1 (FA decarboxylase) (Goncalves et al., 2016). This can be seen in Table 1.
2. The optimum temperature, pH, and sugar content for biotransformation was: glucose 9 gL^−1^; maltose 86 gL^−1^; fructose 5 gL^−1^; (pH 5) at 35°C. Since FA, is the starting material in the synthesis of vanillin and other aromatic compounds, such as vanillic acid, vanillin, and vanillic alcohol (Kumar and Pruthi, 2014; Tanruean and Rakariyatham, 2016), we have tried to find these substances in our experiments but without success. Probably the enzymes for the subsequent biotransformation of 4-VG to vanillin were absent.
3. The highest conversion of pure FA to 4-VG was obtained by SC (66.27%), while Prelude had the lowest (23.71%).

This is in line with the conclusion of Watanabe et al. (2009).

When flax seeds were added to medium A, 4-VG is produced. In these fermentations, β-glucosidase was added. With this, the highest amount of 4-VG was produced by SC when golden flax seeds (0.732 mgL^−1^) were used, while the non-*Saccharomyces* spp. produced considerably lower amounts (0.003–0.285 mgL^−1^).

### The Effect of Flax Seeds and PEF in Beer Wort

Table 2 shows the results from the Control and PEF fermentations, while β-glycosidase was added again. An increase in the concentration of 4-VG was also observed. All yeasts proved capable of producing 4-VG, when FA was available from wort and flax seeds during fermentation: in this process, the first condition is the presence of the enzyme, which is added to liberate from the β-glucosidic form the covalently bonded forms of FA derivatives, or to neutralize the very tiny amounts of cyanate ions from flax seeds. Given independent-samples, the one-tailed *t*-test was conducted to compare the concentration of 4-VG both with the enzyme and without it during fermentation. The only factor that was checked during the *t*-test was the enzyme, so the results concern Control and PEF fermentations, and each variety of flax seeds. There was not a significant difference in the concentrations of 4-VG for any yeast. The results of the *t*-test are presented in Table 2.

**Table 2:** Results of control and PEF fermentations with brown and gold flax seeds in beer wort: Concentration of 4VG (mgL^−1^) with the presence of β-glycosidase (+) and absence (-).

**4VG**		**Brown flax seed**		**Gold flax seed**		***t*-test**
		**PEF**	**Control**	**PEF**	**Control**	
*Mets*	(–)	10.09 ± 2.11	8.44	13.99 ± 2.74	3.12	0.22
	(+)	11.49 ± 2.76	12.58	15.81 ± 3.52	5.62	
*Prelude*	(-)	10.17 ± 1.99	6.26	10.78 ± 2.05	16.08	0.24
	(+)	11.45 ± 1.14	6.58	16.66 ± 3.73	15.73	
*Biodiva*	(-)	10.81 ± 1.82	4.86	10.09 ± 0.45	9.34	0.23
	(+)	16.06 ± 4.31	3.52	10.92 ± 1.99	9.73	
*S.C*.	(–)	11.90 ± 0.65	5.91	11.83 ± 2.45	6.94	0.46
	(+)	9.19 ± 0.56	4.54	15.63 ± 3.83	7.88	

**Figure 1:**
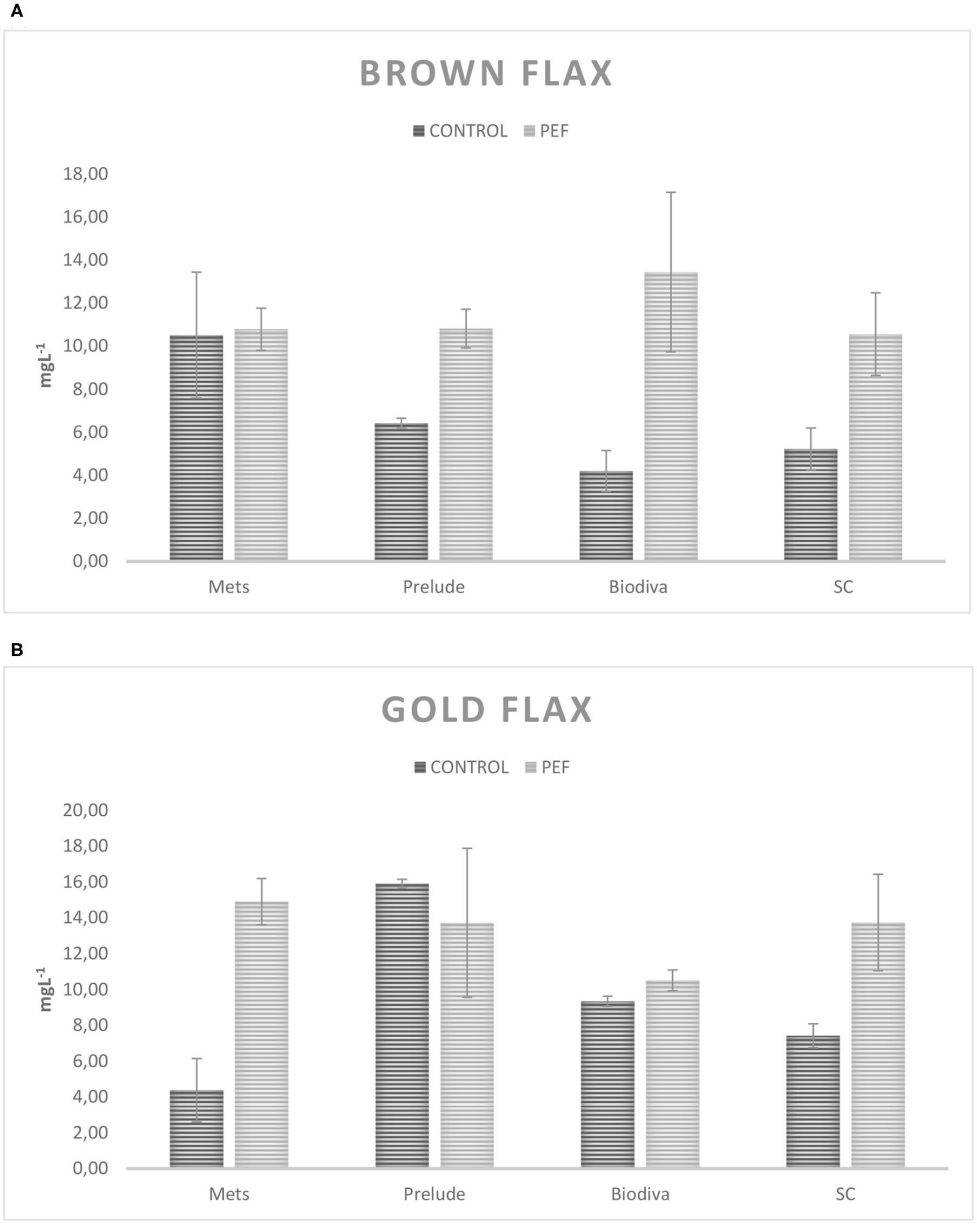
Differences in the concentration of 4VG (mgL^−1^) among PEF fermentations and control: **(A)** brown flax seed variety, **(B)** gold flax seed variety. Error bars represent SD.

Since there is no significant difference concerning the presence of the enzyme, averages of the fermentations with and without β-glycosidase are shown for each yeast in Table 3. In this table, it is obvious that PEF treatment in the substrates before fermentation resulted in an increased production yield of 4-VG, by an average of 120%. Specifically, to evaluate the differences due to PEF, the average PEF fermentations compared to Control fermentations and the results are presented in Figures 1A,B. It appears that the gold flax variety produces better results in 4-VG from PEF fermentations. For yeasts, SC had more stable results and presented a difference between Control and PEF fermentations for flax seed varieties. Independent-samples and a one-tailed *t*-test were performed, without considering yeast strains for assessing the statistical difference of the concentration of 4-VG, with and without PEF treatment in the substrates. The results show a significant difference (*p* = 0.0005).

**Table 3:** Concentration of 4VG (averages and SD) in mgL^−1^ without the presence of β-glycosidase.

**4VG**	**Brown flax seed**					
	**Control**		**Average**	**PEF**		**Average**
*Mets*	8.44	12.58	10.51 ± 2.93	10.09	11.49	10.79 ± 0.98
*Prelude*	6.26	6.58	6.42 ± 0.22	10.17	11.45	10.81 ± 0.90
*Biodiva*	4.86	3.52	4.19 ± 0.95	10.81	16.06	13.43 ± 3.71
*S.C*.	5.91	4.54	5.22 ± 0.97	11.90	9.19	10.55 ± 1.92
	**Gold flax seed**					
	**Control**		**Average**	**PEF**		**Average**
*Mets*	3.12	5.62	4.37 ± 1.77	13.99	15.81	14.90 ± 1.29
*Prelude*	16.08	15.73	15.90 ± 0.25	10.78	16.66	13.72 ± 4.16
*Biodiva*	9.34	9.73	9.53 ± 0.28	10.09	10.92	10.50 ± 0.58
*S.C*.	6.94	7.88	7.41 ± 0.67	11.83	15.63	13.73 ± 2.69

When the external electric field is applied to the must enriched in flax, a critical electric potential across the cell membrane is induced. This potential causes a profound modification of the cell membrane which is rather mechanical. Consequently, the permeability of the membrane increases considerably, pores are formed simultaneously and release FA derivatives which are localized to the internal part of the cell. In our case, the disorganization occurs on the wort and on the flax seed. The FA derivatives have further been decarboxylated from yeast enzymes to yielding higher amounts of 4-VG.

## Advantages of the Method

### Modulation of the Phenolic Flavor

There are many influences on the formation of phenols in beer: the proportion of wheat malt in the grist, the mashing conditions, and wort boiling, as well as the fermentation procedure, yeast strain, and contamination presence (Bartolome et al., 1996). Decarboxylation of flavor-inactive phenolic acids with a high flavor threshold like FA in 4-VG was viable until now in two ways: high temperature treatment during the beer production process or by enzymatic decarboxylation in fermentations (McMurrough et al., 1996). Both, however, present disadvantages. With the proposed method, the phenolic flavor can be modulated by increasing or decreasing the time of PEF treatment as in the case of extraction of alpha acids from hops (Ntourtoglou et al., 2020), or by controlling the quantity of flax seeds.

Modulating the phenolic flavor serves another purpose. An equilibrium must be respected between enhancement of aroma complexity and its phenolic off-flavor character. Indeed, 4-VG is an ingredient of phenolic off-flavors (Zhu and Cui, 2013), which are possibly one of the unwanted compounds in beer production. Phenolic off-flavors have a low flavor threshold (0.2–0.4 mgL^−1^) and are characterized by a clove and medical aroma, highly undesirable in most beers (Mertens et al., 2017). Yet it is essential for the flavor of some wheat beer styles; *S. cerevisiae* brewing yeasts are used in the fermentation of beers in which a clove-like aroma is actually desired (Vanbeneden and Gils, 2008; Goncalves et al., 2016). The choice of the yeast, the conditions of PEF treatment, the quantity of flax seeds, are parameters of this method easily adapted to each beer style.

### Enhancement of Antioxidant Capacity

Naturally-occurring phenols in malt (germinated barley) have proven their antioxidant activities. Although FA is potentially a good antioxidant of beer, its action is limited due to low concentration in free form. Any pathway or physicochemical method that involves hydrolysis of cell wall feruloylated polysaccharides to the free form of FA during mashing and kilning is suitable to improve the natural antioxidant capacity of the wort, as well as the beer obtained from it. From this view, the proposed technology enhances the antioxidant capacity of the wort or the beer.

### Extension of the Work to Other Cell-Wall Degrading Enzymes

FA is mainly esterified to arabinofuranosyl residues of heteroxylans in barley cell walls (Mathew and Abraham, 2006). FA esterases can release FA from feruloylated plant cell wall polysaccharides, enhanced by the presence of cell-wall degrading enzymes (Bartolome et al., 1996). Research shows that when mashing, decarboxylation of FA is optimal.

Attempts have been made to obtain FA enzymatically, which is difficult due to covalent bonds between FA and biopolymers in plant cell walls (Fincher and Stone, 1986). It has been shown that enzymes from *Streptomyces*, e.g., acetyl xylan esterase and other xylanases, are used for enzymatic production of FA from defatted rice bran, suggesting extraction of FA from sources like corncob, raw rice bran, or wheat bran.

Disorganization of the plant cell wall using PEF will provide access to enzymes to target substrates releasing precursors convertible into desired compounds and enhancing the bio-flavoring.

## Conclusions

In 15 min, PEF treatment of beer wort, supplemented with flax seeds before fermentation, yields 4-VG production efficiencies up to 120% (mgL^−1^). The wort was supplemented with a β-glycosidase, which is positive in terms of productivity in combination with PEF. The treatment chamber is box type, composed of two rectangular parallel stainless steel electrodes. Voltage applied is 1 kV with pulses at 900 × 10^3^. Treatment efficacy is independent of the fermentation yeast. In our work, four commercial strains of non-*Saccharomyces* and one *Saccharomyces* were used.

## Data Availability Statement

All datasets presented in this study are included in the article/supplementary material.

## Author Contributions

ET: main experiment, analysis of volatile compounds, writing, and editing the body text. GN: pulsed electric field design, construction, and main experiment. FD: analysis of volatile compounds and fermentations. PT: microorganisms culture and editing the body text. SL: editing the text and pulsed electric fields. TD: chemical analysis of volatile compounds. VD: designed the study and editing the body text. All authors contributed to the article and approved the submitted version.

## Conflict of Interest

The authors declare that the research was conducted in the absence of any commercial or financial relationships that could be construed as a potential conflict of interest.

## Abbreviations

PEF: Pulsed Electric Field
FA: Ferulic Acid
4-VG: 4-Vinylguaiacol
t_i_: Peak time duration
t_p_: Pause time duration.
